# A luminescent-based protocol for NAD^+^/NADH detection in *C. elegans*, mice, and human whole blood

**DOI:** 10.1016/j.xpro.2024.103428

**Published:** 2024-11-01

**Authors:** He-Ling Wang, Jianying Zhang, Shu-qin Cao, Maria Jose Lagartos-Donate, Shi-qi Zhang, Sofie Lautrup, Zeping Hu, Costas A. Lyssiotis, Riekelt H. Houtkooper, Evandro F. Fang

**Affiliations:** 1 Department of Clinical Molecular Biology, University of Oslo and Akershus University Hospital, 1478 Lørenskog, Norway; 2 Xiangya School of Stomatology, Central South University, Changsha, Hu’nan 410083, China; 3 School of Pharmaceutical Sciences, Tsinghua University, Beijing, China; 4 Department of Molecular & Integrative Physiology, University of Michigan, Ann Arbor, MI 48109, USA; 5 Department of Internal Medicine, Division of Gastroenterology and Hepatology, University of Michigan, Ann Arbor, MI 48109, USA; 6 Rogel Cancer Center, University of Michigan, Ann Arbor, MI 48109, USA; 7 Laboratory Genetic Metabolic Diseases, Amsterdam UMC Location University of Amsterdam, Meibergdreef 9, Amsterdam, the Netherlands; 8 Amsterdam Gastroenterology, Endocrinology and Metabolism, Amsterdam, the Netherlands; 9 Amsterdam Cardiovascular Sciences, Amsterdam, the Netherlands; 10 The Norwegian Centre on Healthy Ageing (NO-Age) and the Norwegian National Anti-Alzheimer’s Disease (NO-AD) Networks, Oslo, Norway

**Keywords:** Cell Biology, Metabolism, Molecular Biology, Neuroscience

## Abstract

Here, we present a NAD^+^/NADH detection assay for evaluating NAD^+^, NADH, and NAD^+^/NADH ratio across diverse biological models, including *Caenorhabditis elegans*, mouse muscle tissue, mouse whole blood, and human whole blood. We describe steps for sample collection and preparation from different models as well as detection and calculation of NAD^+^ and NADH levels. This protocol is applicable for quantifying cellular/tissue NAD^+^ and NADH levels across different biological models.

## Highlights

• Instructions for affordable detection of NAD^+^ and NADH in large sample sizes
• Steps to detect NAD^+^/NADH in *C. elegans*, mice, and human blood samples

## Abstract (editor highlights)

Publisher’s note: Undertaking any experimental protocol requires adherence to local institutional guidelines for laboratory safety and ethics.

## Graphical abstract

**Figure:**
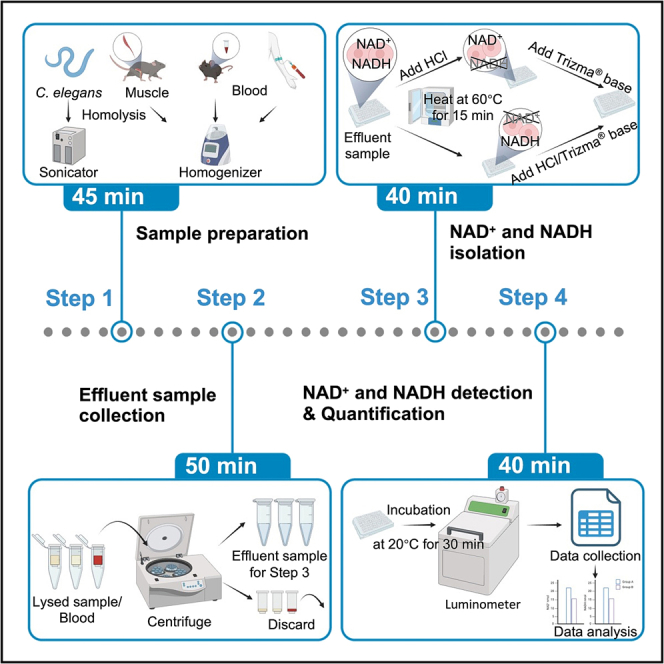

## Before you begin

Nicotinamide adenine dinucleotide (NAD), which includes the oxidized form NAD^+^ and the reduced form NADH, is a fundamental molecule in life and health. NAD orchestrates cellular redox reactions and cellular pathways that directly regulate energy metabolism, Ca^2+^ signaling, neuronal plasticity, cellular resilience, to ageing and longevity.^1^^,^^2^^,^^3^^,^^4^^,^^5^ As an electron acceptor, NAD^+^ is critical for energy metabolism and cellular processes such as glycolysis, citric acid synthesis, and β-oxidation.^6^^,^^7^ Meanwhile, NADH acts as an electron carrier during oxidative phosphorylation (OXPHOS), facilitating electron transfer.^6^ The dynamic interconversion between NAD^+^ and NADH maintains cellular redox balance and influences various physiological processes.^8^ While the ratio of NAD^+^/NADH reflects cellular redox balance significantly; the absolute levels of NAD^+^ in cells is equally important^9^ since it directly impact an organism’s capacity to generate energy and maintain metabolic homeostasis.^5^ Furthermore, while normal ageing leads to a reduction of NAD^+^, which accelerates ageing itself, supplementation with different precursors of NAD^+^, such as nicotinamide riboside (NR) or nicotinamide mononucleotide (NMN), can effectively boost levels of this molecule and ameliorate disease pathologies along with the ageing process observed in worms and mice.^8^^,^^10^^,^^11^^,^^12^^,^^13^^,^^14^^,^^15^

It is therefore important to detect changes in the levels of NAD^+^ and NADH in cells, tissues, and bio-fluids from individuals with disease and at different ages as well as in the conditions before and after interventions. The alteration in NAD^+^ levels (and the NAD^+^/NADH ratio) can serve as mechanistic exploration and interventional endpoints under different circumstances. While accurate detection of NAD^+^ and related metabolites has been established using different mass spectrometry methods,^4^^,^^11^^,^^13^ the high technical requirements and cost limitations hinder its widespread application worldwide. To overcome this limitation, we utilized a commercially available NAD^+^/NADH detection kit and conducted in-house modification and optimization to enable the detection of NAD^+^/NADH in various tissues/cells with minimal sample requirements. In this protocol, three distinct models were employed: *C. elegans* (the wild type/WT strain known as N2), C57BL/6 mice subjected to conditions with or without NR supplementation, and whole blood samples from humans.

***Note:*** In each stage of the experiment, the duration of each step should be adjusted in accordance with the total number of groups involved. Here, the time for each experiment was calculated based on specific numbers of groups: 8 samples for *C. elegans,* 6 samples for mouse muscle, and 6 samples for mouse whole blood.

### Institutional permissions

Human experiments in this study were approved by Regional Committees for Medical and Health Research Ethics (REK), under application number 273644. All animal experiments in this study were in accordance with the Norwegian Law for Protection of Animals and approved by local animal ethics committee (FOTS ID 29730).

### Preparation of *C. elegans* sample

**Timing: 45 min**

This experiment is designed for the detection of whole-body NAD^+^ and NADH levels in adult Day 1 worms with/without one-day NR pretreatment. Due to the sensitivity of the method, a minimum of 600 adult worms per group is needed. If one aims to detect NAD^+^/NADH in larval worms (L1 to L4), higher numbers might be needed. For *C. elegans* maintenance and culturing, please see published protocols.^10^^,^^16^^,^^17^

These steps enable the collection and preparation of *C. elegans* samples, ensuring they are clean, appropriately weighed, and consist of synchronized populations of worms. Subsequently, the worms are lysed using a sonicator, culminating in the detection of NAD^+^ and NADH.

1. Pick a 1.5 mL microcentrifuge tube, weigh it, and record the weight as W1.
2. Collect well-fed (*ad libitum*) synchronized adult Day 1 *C. elegan*s (in the Fang Laboratory, the day after L4 is defined as adult Day 1) from the maintenance nematode growth media (NGM) plate(s):
  a. To add 1.5 mL of M9 buffer (see materials and equipment) to each plate, wash the worms from the NGM plate, and collect the worm-containing buffer in a 1.5 mL tube.
  b. Allow the worms to settle in the bottom of the tube by gravity for 1–2 min and then remove the supernatant.
  c. Resuspend the worms with 1 mL of M9 buffer to clear away bacteria, then remove the supernatant.
  d. Repeat steps ‘b’ and ‘c’ three times until the supernatant is free of bacteria.

***Note:*** Collect a minimum of 600 adult worms (*e.g.* 250 worms/10-cm plate, pooled from three plates). The age of the worms may vary from the experimental objectives.

3. Vacuum dry the 1.5 mL tube to evaporate residual liquid using a centrifugal vacuum concentrator:
  a. Keep the lid open.
  b. Vacuum the tube for 10 min at 4°C.
4. Weigh and record the tube containing samples as W2.
5. Calculate the weight of worms and get ready for lysis. The weight of worms is equal to (W2 - W1).

***Note:*** The samples are now ready to be lysed.

**CRITICAL:** If frozen samples are used for NAD^+^/NADH detection, avoid multiple freeze and thaw cycles to preserve sample integrity and stability. Freezing the sample might result in a lower detected amount of NAD, and therefore all samples that should be compared have to be treated the same way.

6. Homogenization of worms:
  a. Add 400 μL of ice-cold 1x PBS in each of the worm-containing 1.5 mL tube.
  b. Homogenize the worms in a sonicator at high power for ten cycles. The tubes should be precooled down on ice and always kept always on ice to enable the deactivation of NAD^+^-consuming enzymes (Bioruptor Plus sonicator, Diagenode).
    i. Set the sonicator to −8°C.
    ii. Homogenize the worms at high power for ten cycles. For one cycle: 30 s ON, 30 s OFF.

***Note:*** Alternatively, homogenize the sample using a homogenizer (*e.g.*, FastPrep-24 5G).

7. Collect NAD^+^/NADH-containing sample solution:
  a. Transfer the lysed worms into pre-cooled 10 kDa spin columns (Cat.# MRCPRT010) for the deproteinization of biological samples.
  b. Centrifuge the spin column at 13,000 × *g* at 4°C for 20 min.
  c. Effluents after centrifuge are collected and kept on ice for NAD^+^ and NADH detection.

**CRITICAL:** The aforementioned steps (1–7) describe the sonication of *C. elegans* sample using a Diagenode Bioruptor Plus. Different sonicators may be utilized; however, adjustments to these steps may be necessary based on the specific instrument being used.

### Preparation of mouse muscle samples

**Timing: 45 min**

These steps show the collection and preparation of mouse muscle tissue, ensuring homogenization of the sample using a sample disruption instrument and preparing the sample for NAD^+^/NADH detection.

8. Animal sacrifice:
  a. Conduct the procedure in accordance with local ethical guidelines and approved animal protocols.
  b. Place the mouse in an induction chamber and euthanize using CO_2_ inhalation following institutional guidelines.
  c. Confirm the depth of anesthesia by checking the pedal reflex.

***Note:*** As CO_2_ may affect the function of some subcellular organs, such as mitochondria, thus an alternative sacrifice approach is via cervical dislocation (if ethical approval is possible in related institution).

9. Tissue dissection:
  a. Perform dissection under sterile conditions.
  b. Spray the body with 70% ethanol to sterilize the area of operation.
  c. Use iris scissors and forceps to carefully expose and remove the skin from the left leg, revealing the leg muscle.
  d. Identify the gastrocnemius and dissect it.

***Note:*** Make sure to exclude the tendons.

10. Tissue harvesting:
  a. Use clean and sterile scissors and forceps to cut the muscle tissue to small pieces while minimizing potential damage to adjacent structures. Place the harvested tissue in a pre-chilled sterile petri dish with PBS.

***Note:*** Keep all equipment cold in dry ice before the process and work fast during in the dissection. This is especially important if NAD^+^/NADH detection procedure is not applied immediately, *e.g.*, one may want to collect many other tissue samples before starting NAD^+^/NADH detection. These samples should be snap-frozen as described below after step 11.

11. Tissue preservation:
  a. Pick a 1.5 mL tube, weigh it and record the weight as W1.
  b. Wash the gastrocnemius with PBS twice.
  c. Place the tissue in the 1.5 mL tube, weigh it and record the weight as W2.
  d. Calculate the weight of sample, and the mass of gastrocnemius tissue is equal to (W2 - W1).

***Note:*** Snap-freeze the tissue in the 1.5 mL tube in liquid nitrogen if the NAD^+^/NADH detection is not performed immediately after the dissection.

**CRITICAL:** If frozen samples are used for NAD^+^/NADH detection, avoid multiple freeze and thaw cycles to preserve sample integrity and stability. Freezing the sample might result in a lower detected amount of NAD, and therefore all samples that should be compared have to be treated the same way.

12. Homogenization of gastrocnemius:
  a. Transfer 50 mg of the gastrocnemius tissue from the 1.5 mL tube to an ice-cold lysis matrix D tube with 400 μL of 1x PBS.
  b. Homogenize the sample using a bead-based homogenizer (The FastPrep-24 5G). Parameters were set up as follows: speed = 6.0 m/s, adaptor = QuickPrep, time = 40 s, lysis matrix as D, 3 cycles, pause time = 10 s.
13. Collection of effluent sample:
  a. Centrifuge the homogeneous mixture at 13,000 × *g* at 4°C for 10 min.
  b. Transfer the supernatant to an ice-cold 10 kDa spin column (Cat.# MRCPRT010) for the deproteinization of biological samples.
  c. Centrifuge the tube at 13,000 × *g* at 4°C for 30 min.
  d. Keep the effluent sample on ice for NAD^+^ and NADH detection.

***Note:*** Repeat step 13 to achieve sufficient supernatant of homogeneous mixture if needed.

### Preparation of mouse whole blood sample

**Timing: 45 min**

These steps outline the collection and preparation of mouse whole blood from the carotid artery and the collection of the effluent sample for NAD^+^/NADH detection.

14. Collect an appropriate volume of blood from carotid artery:
  a. Remove all food sources from the mice cages exactly 12 h prior to the scheduled blood collection. Ensure continuous access to water throughout the fasting period to maintain hydration, unless the experimental protocol specifies otherwise.
  b. Begin by anesthetizing the mouse to ensure it is immobile and insensate.
  c. Check for the absence of reflexes to confirm the depth of anesthesia.
  d. Perform decapitation to facilitate blood collection.
  e. Use a syringe to collect blood directly from the carotid artery as it bleeds. Aim for a blood volume of 100–200 μL and transfer it into a labeled EDTA tube immediately.
  f. Immediately freeze the collected blood sample in liquid nitrogen to preserve its integrity for NAD^+^/NADH detection.

***Note:*** For step 14e, a cardiac puncture, retro-orbital blood collection, or other methods that allow for minimal blood volume may also be effective. The origin of the blood sample is not expected to significantly impact the results.

**CRITICAL:** If frozen samples are used for NAD^+^/NADH detection, avoid multiple freeze and thaw cycles to preserve sample integrity and stability. Freezing the sample might result in a lower detected amount of NAD, and therefore all samples that should be compared have to be treated the same way.

15. Lyse the cells of whole blood:
  a. Transfer 100 μL of the whole blood from the EDTA tube to an ice-cold lysis matrix D tube.
  b. Homogenize the sample using a bead-based homogenizer (The FastPrep-24 5G).
    i. Set up the speed at 6.0 m/s, adaptor as QuickPrep, time at 40 s, lysis matrix as D, cycle as 3, pause time as 10 s.
  c. Centrifuge the tube at 13,000 × *g* at 4°C for 10 min.
16. Collect effluent sample:
  a. Transfer the supernatant from step 15c into an ice-cold 10 kDa spin column (Cat.# MRCPRT010) for the deproteinization of biological samples.
  b. Centrifuge the tube at 13,000 × *g* at 4°C for 30 min to filter out hemoglobin and other substances.
  c. Dilute the sample to a 1:3 volume ratio with 1x PBS (the dilution factor = 4) and mix gently, i.e., combine 50 μL of blood supernatant with 150 μL of PBS.
  d. Keep the diluted sample on ice for NAD^+^ and NADH detection.

***Note:*** Step 16a-b is essential to get rid of NAD^+^-consuming proteins in different cells as well as to eliminate the ‘red-colored’ hemoglobin substances).

### Preparation of human whole blood sample

**Timing: 45 min**

These steps outline the collection and preparation of human whole blood from the median cubital vein, as well as the collection of the effluent sample for NAD^+^/NADH detection.

17. Collect an appropriate volume of blood median cubital vein:
  a. Before drawing the blood, confirm the participant’s identity and review any relevant medical history, and ensure that the participant has been fasting before blood withdrawal.
  b. Prepare the venipuncture site, median cubital vein, by cleaning it thoroughly.
  c. Apply a tourniquet above the site.
  d. Insert a sterile needle into the vein (30-gauge needle) at a 15°–30° angle.
  e. Collect the required blood volume (5 mL) into a labeled EDTA tube.
  f. Remove the tourniquet and withdraw the needle, applying pressure to the site.
  g. Secure the puncture site with gauze and adhesive tape once bleeding stops.
  h. Label and invert the tube gently to mix.

***Note:*** Within 2 min store samples at −80°C if not used immediately, until processing.

**CRITICAL:** If frozen samples are used for NAD^+^/NADH detection, avoid multiple freeze and thaw cycles to preserve sample integrity and stability. Freezing the sample might result in a lower detected amount of NAD, and therefore all samples that should be compared have to be treated the same way.

18. Lyse the cells of whole blood:
  a. Transfer 200 μL whole blood from the EDTA tube to an ice-cold lysis matrix D tube.
  b. Homogenize the sample using a bead-based homogenizer (The FastPrep-24 5G).
    i. Set up the speed at 6.0 m/s, adaptor as QuickPrep, time at 40 s, lysis matrix as D, cycle as 3, pause time as 10 s.
  c. Centrifuge the tube at 13,000 × *g* at 4°C for 10 min.
19. Collect effluent sample:
  a. Transfer the supernatant from step 18c into an ice-cold 10 kDa spin column (Cat.# MRCPRT010) for the deproteinization of biological samples.
  b. Centrifuge the tube at 13,000 × *g* at 4°C for 30 min to filter out hemoglobin and other substances (this step is essential as to get rid of NAD^+^-consuming proteins in different cells as well as to eliminate the ‘red-colored’ hemoglobin substances).
  c. Dilute the sample to a 1:3 volume ratio with 1x PBS (the dilution factor = 4) and mix gently, i.e., combine 50 μL of blood supernatant with 150 μL of PBS.
  d. Keep the diluted sample on ice for NAD^+^ and NADH detection.

***Note:*** If the blood is very thick, *e.g.*, from elderly donors, 100 μL blood can be diluted with 100 μL cold 1x PBS prior to running it through the columns. This will prevent clotting of the columns. In step 19c, adjust the dilution to 1:1.5 to ensure all samples have the same dilution factor, *i.e.*, combine 100 μL of diluted blood supernatant with 150 μL of PBS.

### Preparation of reagents using the NAD/NADH-Glo assay kit

**Timing: 20–30 min**

20. Preparation of reconstituted luciferin detection reagent (RLDR) following the manufacturer’s instructions with modifications:
  a. Thaw the reconstitution buffer in the NAD/NADH-Glo Assay Kit.
  b. Equilibrate the reconstitution buffer and lyophilized luciferin detection reagent to 25°C.
  c. Transfer the entire content of the reconstitution buffer bottle to the amber bottle of lyophilized luciferin detection reagent.
  d. Mix by swirling or inversion to obtain a uniform solution.
  e. Aliquot reconstituted luciferin detection reagent into 15 mL centrifuge tubes, with 5 mL per tube.
  f. The solution can be stored at −20°C up to 3 months.

***Note:*** Do not vortex or shake vigorously.

21. Preparation of reconstitute NAD cycling enzyme:
  a. Reconstitute one vial of NAD cycling enzyme by adding 275 μL of ddH_2_O.
  b. Mix by gently swirling the vial.
  c. Aliquot NAD cycling enzyme into 1.5 mL tubes, with 20 μL per tube.
  d. Store at −20°C for future usage. It can be freeze-thawed and reused up to twice.

***Note:*** Store the Reductase Substrate at −80°C. Minimize multiple freeze-thaw cycles of all reagents by making several aliquots. All solution of reagents remains effective for a period of 3 month upon opening as indicated by the product manual.

## Key resources table

**Table:** 

REAGENT or RESOURCE	SOURCE	IDENTIFIER
**Bacterial and virus strains**		

*E. coli* (OP50)	Caenorhabditis Genetics Center (CGC)	OP50

**Chemicals, peptides, and recombinant proteins**		

Potassium dihydrogen phosphate, KH_2_PO_4_	Sigma-Aldrich	Cat.# 1.04871
Disodium hydrogen phosphate, Na_2_HPO_4_	Sigma-Aldrich	Cat.# 1.06585
Sodium chloride, NaCl	Sigma-Aldrich	Cat.# S9888
Magnesium sulfate, MgSO_4_	Sigma-Aldrich	Cat.# M7506
Sodium hypochlorite, NaClO	Sigma-Aldrich	Cat.# 1056142500
Sodium hydroxide, NaOH	Sigma-Aldrich	Cat.# 106498
Potassium chloride, KCl	Sigma-Aldrich	Cat.# P5405
Dodecyltrimethylammonium bromide, DTAB	Sigma-Aldrich	Cat.# D8638
Hydrochloric acid, HCl	Sigma-Aldrich	Cat.# H9892
Trizma base	Sigma-Aldrich	Cat.# T1503
β-Nicotinamide adenine dinucleotide, NAD^+^	Sigma-Aldrich	Cat.# N8285
β-Nicotinamide adenine dinucleotide, NADH	Sigma-Aldrich	Cat.# N6660
Nicotinamide riboside chloride, NR	NIAGEN (ChromaDex)	Cat.# 40C910-19237-21

**Critical commercial assays**		

NAD/NADH-Glo Assay Kit	Promega	Cat.#G9072

**Experimental models: Organisms/strains**		

*C. elegans*: N2 (wild-type Bristol isolate)	CGC	N/A
Mouse: C57BL/6 (wild type)	The Section for Comparative Medicine (KPM) at the University of Oslo	N/A
Human blood	Collected in UiO/Ahus with ethical approval	N/A

**Software and algorithms**		

Omega	BMG LABTECH	Version 3.00 R2
Excel	Microsoft Excel	www.microsoft.com/en-us/microsoft-365/excel
GraphPad Prism Version 10.1.1	GraphPad	www.graphpad.com

**Other**		

Petri dishes, 92 × 16 mm	Sarstedt	Cat.# 82.1473.001
1.5 mL tube	Sarstedt	Cat.# 72.706
15 mL centrifuge tube	Sarstedt	Cat.# 62.554.502
50 mL centrifuge tube	Sarstedt	Cat.# 62.547.254
10 kDa spin column	Millipore	Cat.# MRCPRT010
Lysing Matrix D, 2 mL tube	MP Biomedicals	SKU: 116913050-CF
96-well luminometer plates	Corning	Cat.# 3917
Test tube shaker (vortexer)	Heidolph	Cat.# 05514
Microplate shaker	VWR	PMS-1000i
Microcentrifuge	Thermo Scientific	Cat.# 75002402
Stereo microscope	Zeiss	Cat.# 495009-9880-010
Cooled incubator	PHC	MIR-554-PE
Incubation shaker	Infors HT	Ecotron
Water bath sonicator	Diagenode	Bioruptor Plus
Centrifugal vacuum concentrator	Eppendorf (Merck)	Model 5301
Bioruptor Plus sonicator	Diagenode	B0102000
The FastPrep-24 5G (homogenizer)	MP Biomedicals	SKU: 116005500
Luminometer	BMG LABTECH	LUMIstar Omega

## Materials and equipment

**Table:** M9 buffer

Reagent	Final concentration	Amount
NaCl	5 mg/mL	5 g
Na_2_HPO_4_	6 mg/mL	6 g
KH_2_PO_4_	3 mg/mL	3 g
ddH_2_O	N/A	up to 1 L
**Total**	**N/A**	**1 L**

Sterilize by autoclaving. Add 1 mL of 1 M MgSO_4_ after autoclaving. Store at 20°C.

**Table:** Bleaching buffer

Reagent	Final concentration	Amount
NaOH (5 N)	0.5 N	1 mL
NaClO (5%)	1%	2 mL
ddH_2_O	N/A	7 mL
**Total**	**N/A**	**10 mL**

Store bleaching buffer for up to a week at 20°C.

**Table:** 1x PBS buffer

Reagent	Final concentration	Amount
NaCl	8 mg/mL	8 g
Na_2_HPO_4_	1.44 mg/mL	1.44 g
KCl	0.2 mg/mL	0.2 g
KH_2_PO_4_	0.245 mg/mL	0.245 g
ddH_2_O	N/A	up to 1 L
**Total**	**N/A**	**1 L**

Adjust solution to pH = 7.4. Sterilize by autoclaving. Store at 20°C.

**Table:** DTAB stock solution

Reagent	Final concentration	Amount
DTAB	20%	10 mL
ddH_2_O	N/A	40 mL
**Total**	**N/A**	**50 mL**

Warm the solution in a 37°C water bath to completely solubilize the DTAB. Store at 4°C for up to one month.

**Table:** Base solution

Reagent	Final concentration	Amount
NaOH (1 N)	0.2 N	10 mL
ddH_2_O	N/A	40 mL
**Total**	**N/A**	**50 mL**

No pH adjustment is required. Store at 20°C for up to one month.

**Table:** Base solution with 1% DTAB

Reagent	Final concentration	Amount
DTAB (20%)	1%	2.5 mL
NaOH (0.2 N)	0.19 N	47.5 mL
**Total**	**N/A**	**50 mL**

Store at 4°C for up to one month.

**Table:** Trizma base solution

Reagent	Final concentration	Amount
Trizma base	0.5 M	3.025 g
ddH_2_O	N/A	50 mL
**Total**	**N/A**	**50 mL**

The final pH will be approximately 10.7. Store at 4°C for up to one month.

**Table:** HCl/Trizma solution

Reagent	Final concentration	Amount
HCl (0.4 N)	0.2 N	25 mL
Trizma base (0.5 M)	0.25 M	25 mL
**Total**	**N/A**	**50 mL**

Mix by vortex. Store at 4°C for up to one month.

**Table:** NAD^+^ stock solution

Reagent	Final concentration	Amount
NAD^+^	2 mM	10 mg (1 vial)
PBS buffer	N/A	7.537 mL
**Total**	**N/A**	**7.537 mL**

Aliquot NAD^+^ stock solution into 1.5 mL tubes. Store NAD^+^ stock solution for up to one month at −20°C.

**Table:** NADH stock solution

Reagent	Final concentration	Amount
NADH	2 mM	0.2 mg (1 vial)
PBS buffer	N/A	140.96 μL
**Total**	**N/A**	**140.96 μL**

Aliquot NADH stock solution into 1.5 mL tubes. Store NADH stock solution for up to a month at −20°C.

## Step-by-step method details

### Isolation of NAD^+^ and NADH

**Timing: 40 min**

These steps utilize high temperature (60°C) during the incubation process to selectively eliminate NAD^+^ while preserving NADH. Under acidic conditions, NAD^+^ remains unaffected by elevated temperature, allowing for the targeted removal of NADH. This strategic approach facilitates the separation of NAD^+^ and NADH within the sample for subsequent detection. These steps are following the manufacturer’s protocol of the NAD/NADH-Glo Assay kit.

1. Dispense 50 μL of the effluent sample into the designated well of a 96-well white luminometer plate.
2. Add 50 μL of base solution with 1% DTAB to the designated well.
3. Agitate the plate on a microplate shaker for 2 min.
4. Transfer 50 μL of the sample from the designated well to an empty well, while retaining the remaining 50 μL in the original well.
5. For measuring NAD^+^ and NADH (reduced form NADH is not stable in acidic solution):
  a. Add 25 μL of 0.4N HCl to the original well to convert it into an acid-treated sample.
  b. Do not add 0.4N HCl to the new well to maintain it as a base-treated sample.

***Note:*** NADH is not stable in an acidic solution; NAD^+^ is selectively destroyed by heating in a basic solution.

6. Cover the plate with a sealer and incubate the plate at 60°C for 15 min.
7. Take out the plate from the incubator and allow it to cool down to room temperature for 10 min.
8. For wells measuring NAD^+^, neutralize with addition of 25 μL of 0.5 M Trizma base (wells from step 5a).
9. For wells measuring NADH, add 50 μL of HCl/Trizma base solution (wells from step 5b).
10. Transfer 50 μL of the sample from each well to an empty well for duplication.

### Preparation of NAD^+^ and NADH standards

**Timing: 10 min**

These steps involve the preparation of NAD^+^ and NADH standards through the creating of a dilution buffer, subsequent dilution of stock solutions, and dispensing these standards into a 96-well plate for further analysis.

11. Prepare 6 mL of dilution buffer in a 15 mL centrifuge tube by combining equal volumes (1:1:1:1) of the following four reagents (Use 1.5 mL of each reagent.): PBS, base solution with 1% DTAB, 0.4 N HCl, and 0.5 M Trizma base.
12. Dilute the 2 mM stock solutions of NAD^+^ and NADH separately with the dilution buffer to obtain standard solutions with a concentration of 1 μM.
13. The NAD^+^ and NADH standard working solutions within the range of 0–400 nM can be prepared by referring to the table below:

**Table:** 

Standard #	Volume of 1 μM NAD^+^ or NADH stock standard (μL)	Volume of dilution buffer (μL)	Final concentration (nM)
1	0	200	0
2	1	199	5
3	2	198	10
4	4	196	20
5	8	192	40
6	12	188	60
7	16	184	80
8	20	180	100
9	40	160	200
10	60	140	300
11	80	120	400

***Note:*** The preparation of fresh NAD^+^ and NADH standards is recommended immediately prior to each use.

14. Dispense 50 μL of the respective concentrations of NAD^+^ (or NADH) standard solution into designated wells of a 96-well plate.
15. Replicate each dinucleotide concentration within an assigned well.

### Detection of NAD^+^ and NADH

**Timing: 40 min**

These steps describe the utilization of the commercial NAD/NADH- Glo assay kit to detect the NAD^+^ and NADH, under our optimized conditions.

16. Thaw the NAD/NADH-Glo assay kit reagents on ice and briefly centrifuge the vials with a microcentrifuge.
17. Calculate the total volume required for the mixed solution from the kit:
  a. Use an equal volume of mixed solution as the sample volume (50 μL) for each well.
  b. Prepare the mixed solution following a ratio of reconstituted luciferin detection reagent to NAD cycling substrate to reductase to NAD cycling enzyme to reductase substrate = 200: 40: 1: 1: 1. For instance, to make 2 mL (2000 μL) of the mixed solution, use the following volumes:
     Reconstituted luciferin detection reagent (RLDR): 1800 μL.
     NAD cycling substrate: 360 μL.
     Reductase: 9 μL.
     NAD cycling enzyme: 9 μL.
     Reductase substrate: 9 μL.
  c. Gently invert the solution five times to mix it thoroughly.
    ***Note:*** Ensure that there is enough mixed solution for all wells by preparing at least 500 μL extra (or depending on the total samples being detected). The mixed solution should be prepared immediately before use.
18. Transfer 50 μL of mixed solution to each well, including wells for samples and standard solutions.
19. Gently shake the plate using a plate shaker for 2 min to ensure thorough mixing.
20. Incubate the plate at 20°C for 30 min (incubation time can be further optimized based on signal intensity in pilot experiments conducted in individual laboratories).

***Note:*** Incubation time may vary among different samples.

21. Record luminescence reads using a luminometer (LUMIstar Omega, BMG LABTECH) after incubation:
  a. Perform gain adjustment with the well containing the maximum concentration.

### Data analysis

**Timing: 20 min**

These steps outline the procedures for data analysis.

22. Plot and apply the standard curves using Microsoft Excel and GraphPad Prism (version 10.1.1):
  a. Calculate the average luminescence value for the blank control (zero) standards. Subtract the average blank control standard luminescence value from all other luminescence values to obtain net luminescence values.
  b. Create two standard curves (one for NAD^+^, one for NADH) by plotting the average blank control subtracted luminescence value for each standard concentration (y-axis) against the target dinucleotide concentration (x-axis) of the standard.
  c. Use graphic software to draw a smooth curve through these points to generate the standard curve and perform a linear regression for the entire set of standards.
23. Determine the concentration of NAD^+^ or NADH in the sample by interpolating the net luminescence values against the standard curve.
24. Multiply the resulting value by the appropriate sample dilution factor if used. Corrected concentration = interpolated concentration (from the standard curve) × dilution factor.
25. Normalize the value by dividing it by weight (for *C. elegans* and muscle samples) or volume (for blood samples) of the specific samples:

Normalizedconcentration=correctedconcentration/(Weight(forC.elegansandmuscle)orVolume(forblood))

## Expected outcomes

Detection of NAD^+^ and NADH levels in *C. elegans*, mice, and humans provides important insights into the molecular mechanisms underpinning aging and various diseases, particularly neurodegenerative disorders like Alzheimer’s disease. This in-house protocol employing a commercially available kit provides a reliable and efficient approach for quantifying these essential metabolites. This methodology offers researchers a profound understanding of how NAD^+^ and NADH levels change during an organism’s age or disease progression, making it applicable to diverse model systems.

**Figure 1:**
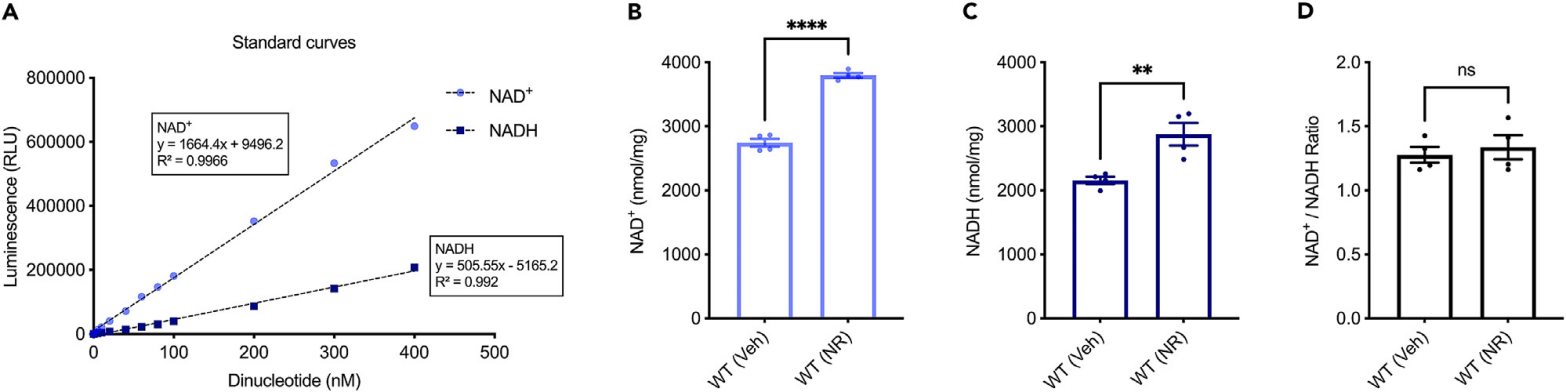
Detection of NAD^+^ and NADH in nematodes (A) Linear range of the NAD/NADH-Glo Assay. Each point represents average luminescence of duplicate reactions measured in relative light units (RLU). Data showing NAD^+^ level (B), NADH level (C) and NAD^+^/NADH ratio (D) in WT *C. elegans* with vehicle (veh) and after 24 h of 2 mM NR treatment (NR). All quantitative data are shown in mean ± S.E.M. from four biological repeats. Student’s *t* test was used for data analysis (ns, no significance; ∗, *p* < 0.05; ∗∗, *p* < 0.01; ∗∗∗, *p* < 0.001).

**Figure 2:**
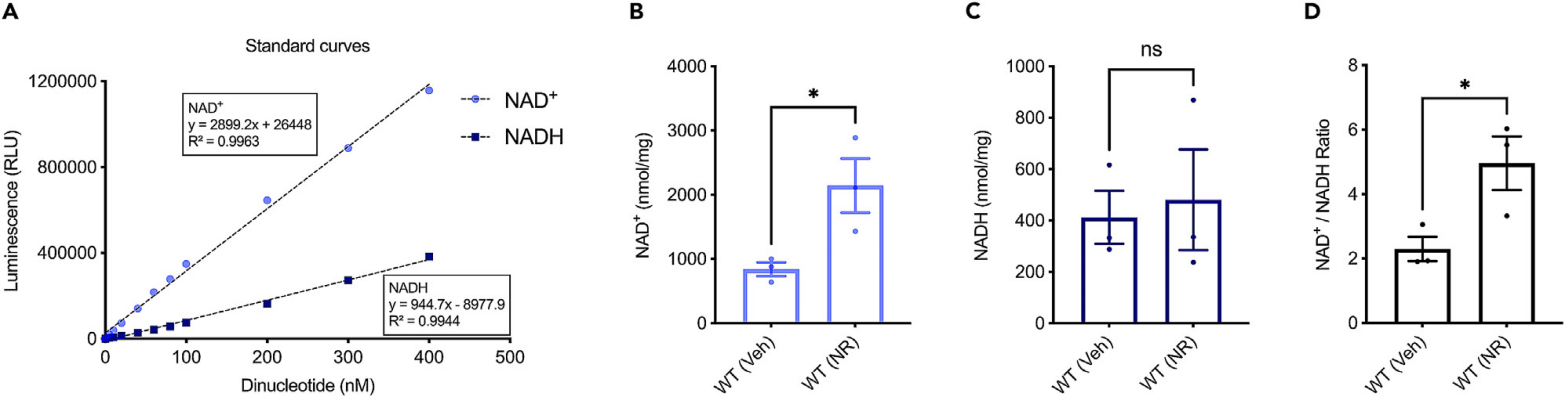
Detection of NAD^+^ and NADH in mouse muscle tissue (A) Linear range of the NAD/NADH-Glo Assay. Each point represents average luminescence of duplicate reactions measured in relative light units (RLU). NAD^+^ level (B), NADH level (C) and NAD^+^/NADH ratio (D) in WT mouse with vehicle (veh) and after 2 weeks of 12 mM NR treatment (NR) are presented. All quantitative data are shown in mean ± S.E.M. from three biological repeats. Student’s *t* test was used for data analysis (ns, no significance; ∗, *p* < 0.05).

**Figure 3:**
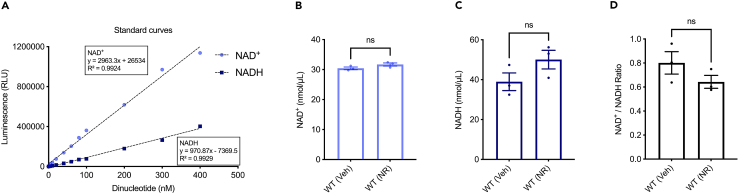
Detection of NAD^+^ and NADH in mouse whole blood (A) Linear range of the NAD/NADH-Glo Assay. Each point represents average luminescence of duplicate reactions measured in relative light units (RLU). NAD^+^ level (B), NADH level (C) and NAD^+^/NADH ratio (D) in WT whole blood with vehicle (veh) and after 2 weeks of 12 mM NR treatment (NR). All quantitative data are shown in mean ± S.E.M. from three biological repeats. Student’s *t* test was used for data analysis (ns, no significance).

**Figure 4:**
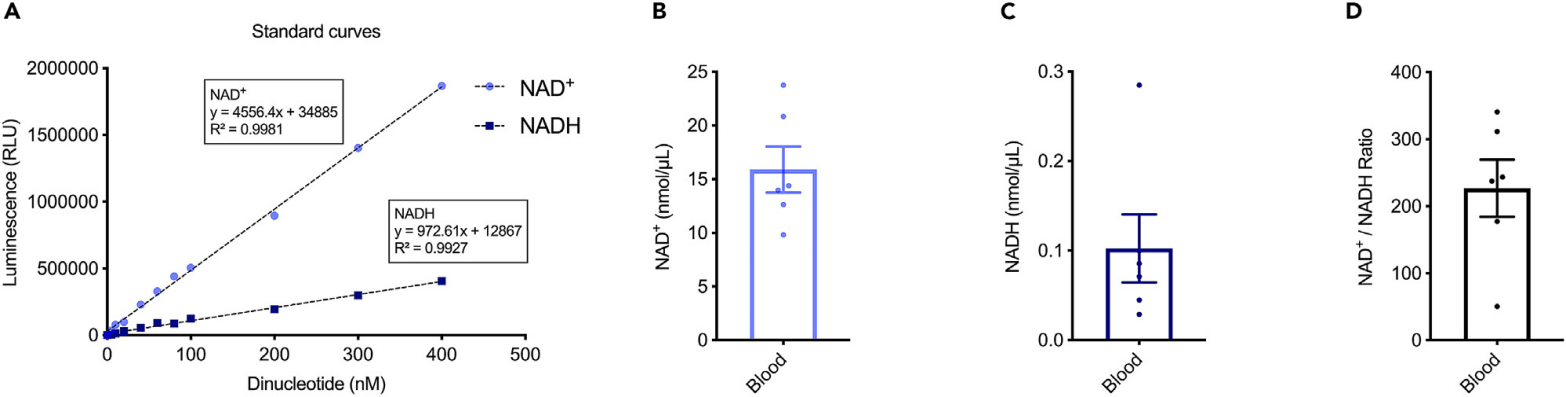
Detection of NAD^+^ and NADH in human whole blood (A) Linear range of the NAD^+^/NADH-Glo Assay. Each point represents average luminescence of duplicate reactions measured in relative light units (RLU). NAD^+^ level (B), NADH level (C) and NAD^+^/NADH ratio (D) in human whole blood. All quantitative data are shown in mean ± S.E.M. from three biological repeats.

In *C. elegans* and mouse models, our results show that NAD^+^, NADH, and the NAD^+^/NADH ratio can be reliably detected. Our method was able to detect blood NAD^+^; however, the NADH levels were mostly under the detection range possibly due to long-term sample storage and other unknown reasons (∗Insert Fixed_Image_Figures 1, 2, 3, and 4 here). These findings underscore the utility of the protocol for comparative studies across different organisms.

Moreover, the protocol’s utility extends to the study of interventions aimed at addressing NAD^+^ changes. For instance, supplementation with an NAD^+^ precursor is known to upregulate cellular NAD^+^ levels, presenting a potential avenue for therapeutic interventions.^4^^,^^8^ By utilizing this protocol, researchers can assess the efficacy of NAD^+^ precursor supplementation in-house in various models (Figures 1, 2, 3, and 4), offering a tool for investigating the impact of such interventions on cellular metabolism and identifying strategies to mitigate age-related and disease-associated NAD^+^ depletion.

Overall, this methodological approach serves as an efficient tool for exploring NAD^+^ dynamics and their implications for aging and various pathological conditions.

## Limitations

While our optimized NAD^+^ and NADH detection protocol utilizing a commercial kit offers a practical and efficient approach, specifically for detecting whole cell/whole tissue levels of NAD^+^ and NADH. However, it is crucial to acknowledge inherent limitations. In addition to monitoring changes in NAD^+^ and NADH in whole cells, researchers are also interested in levels and changes within sub-cellular compartments. However, our protocol is limited to detecting NAD^+^ and NADH in whole cells or tissues and is not ideal for sub-cellular compartment analysis. There are significant ratio differences in NAD^+^/NADH between cytosolic (100:1) and mitochondrial (10:1) compartments that warrant investigation.^18^ Therefore, for studies focusing on subcellular compartments, using a fluorescent reporter, like SoNar, is recommended as an ideal alternative.^18^^,^^19^

Moreover, the specified detection range of 10–400 nM imposes limitations on the kit’s applicability, potentially excluding samples with concentrations outside this range (particularly those with lower NAD^+^ levels; dilution can be applied for samples with higher NAD^+^ before detection) and external validation with mass spectrometry is ideal.^20^^,^^21^ Researchers must exercise caution when dealing with samples near the detection limits to ensure measurement accuracy. For instance, our attempt to utilize the protocol for detecting NAD^+^ and NADH levels in mouse and human brain tissue yielded values falling below the detection range.

Furthermore, the accuracy of sample normalization poses a significant challenge due to potential variations in sample weight that can impact the results. Therefore, maintaining strict attention to sample weights and normalization procedures is imperative for obtaining meaningful and comparable data.

Additionally, the protocol’s exclusive focus on NAD^+^ and NADH may limit its capacity for a comprehensive analysis of the cellular metabolome compared to more advanced methods such as liquid chromatography-mass spectrometry (HPLC-MS).^20^^,^^21^^,^^22^ HPLC-MS methods have been well-developed and allow for simultaneous detection of NAD^+^, NADH, related metabolites, as well as other common metabolites in samples from mouse brain,^11^ muscles from young and older adult humans,^13^ as well as blood and fibroblasts from humans.^4^ These data provide a broader overview of NAD^+^ metabolism, facilitating mechanistic exploration. Moreover, the utilization of isotope-tracer methods for quantifying NAD flux, such as the one developed by Joseph A Baur’s lab,^2^ is essential to investigate intracellular and organismal metabolism (including stability, absorption, bioavailability, and pharmacokinetics, etc.) of different NAD^+^ precursors, such as NR, NMN, and nicotinamide. Researchers should carefully evaluate these limitations against the convenience of the protocol while considering specific research objectives and requirements when selecting the most suitable method for analyzing NAD^+^ and NADH.

Finally, a critical limitation of our current protocol is the management of freeze-thaw cycles, which can significantly compromise sample integrity, particularly concerning NAD^+^ degradation. These cycles risk reactivating NAD-degrading enzymes, leading to unreliable results. Freeze-drying presents an alternative by eliminating the need for thawing and reducing the risk of enzyme activation. However, once the freeze-dried material is rehydrated, the potential for enzyme activity may still persist. We recommend minimizing the number of freeze-thaw cycles and, ideally, using fresh samples to ensure greater reliability.

## Troubleshooting

### Problem 1

The inefficient collection of effluent samples due to inadequate homogenization of muscle tissues and blockage of the spin column (related to steps 12–13 outlined in the “before you begin” section).

### Potential solution

• Inadequate homogenization of muscle tissues.
  ○ Preliminary tissue grinding. The initiation of the homogenization process subsequent to manual grinding of mouse muscle tissue can significantly enhance overall efficiency and efficacy in sample preparation.
  ○ Extend time for homogenization. Prolonging the duration of homogenization allows for better breakdown and mixing of the sample components, thereby improving overall homogeneity.
• Blockage of the spin column.
  ○ Utilize a new 10 kDa spin column for collection. Transfer the remaining sample from the spin column to a fresh pre-cooled spin column, can help optimize the separation and collection of effluent samples, resolving inefficiencies associated with the current column.

### Problem 2

Luminescence reads fall outside of the detection range of 10–400 nM.

### Potential solution

• Samples generating luminescence values greater than that of the highest standard.
  ○ Samples should be further diluted and reanalyzed. We recommend conducting pilot experiments with various dilution factors to determine the appropriate dilution before formal experiments.
  ○ Reduce sample weight of solid tissues can help address values exceeding the max detection range.
• Samples generating luminescence values less than that of the lowest standard (Figure 4C).
  ○ Increase sample weight of solid tissues.
  ○ Adjust the dilution factor to a less dilute form.
  ○ If these adjustments fail to yield values within the desired range, consider using intact whole tissue samples (e.g., entire gastrocnemius or brain) or undiluted samples, to increase the concentration of NAD^+^/NADH.
  ○ Minimize/Avoid the number of freeze-thaw cycles, freeze-drying presents an alternative by eliminating the need for thawing and reducing the risk of enzyme activation.
  ○ In cases where even whole samples result in values below the minimum range, alternative methods such as HPLC/MS or similar techniques should be considered for a more sensitive analysis.

### Problem 3

Large variation of data without meaningful statistical values.

### Potential solution

NAD^+^ exhibits high instability due to rapid consumption by NAD^+^ consumers, such as PARPs, CD38, and sirtuins, as well as susceptibility to exogenous factors like circadian rhythm, fasting, and exercise. To ensure accurate measures of NAD^+^ levels and to enhance data replicability, we propose the following strategies:

• Increase the sample size.
  ○ For *C. elegans*-related studies, it is advisable to include 4–5 biological replicates.
  ○ For mouse samples, both male and female animals should be included with approximately 5–15 mice per gender per group.
  ○ For human samples, the analysis requires a minimum of 10 samples per group (age, gender, disease conditions etc., must be carefully considered during data analysis); identification and exclusion of outliers is also crucial if necessary.
• Implementation of quality control measures.
  ○ Synchronization of samples: For instance, blood samples should be collected at the same time throughout the study, in fasting conditions, and without engaging in any physical exercise. Blood samples should also be flash frozen on liquid nitrogen within 2–5 min after withdrawal.
  ○ To prevent the consumption or degradation of NAD^+^ by other proteins, fresh (or thawed) samples should be kept on ice for all steps unless otherwise specified.
  ○ Care should be taken to avoid overheating the samples during sample homogenization due to the temperature sensitivity of NAD^+^ (which is destroyed at 60°C). Therefore, it is necessary to ensure controlled temperatures during mechanical (or physical) disruption of tissues/cells by always keeping samples on ice and limiting sonication time while allowing sufficient intervals for cooling.

## Resource availability

### Lead contact

Further information and requests for resources and reagents should be directed to and will be fulfilled by the lead contact, Evandro F. Fang (e.f.fang@medisin.uio.no).

### Technical contact

Technical questions on executing this protocol should be directed to and will be answered by the technical contacts, He-Ling Wang (heling.wang@medisin.uio.no) and Jianying Zhang (zhjianying@csu.edu.cn).

### Materials availability

This study did not generate new and unique reagents. All materials, unless otherwise specified, are commercially available with vendors’ information provided.

### Data and code availability

This study did not generate or analyze any additional datasets.

## Acknowledgments

The authors acknowledge the valuable work of the many investigators whose published articles on NAD^+^ and NADH detection they were unable to cite owing to space limitations. This work was supported by the Rosa sløyfe/Norwegian Cancer Society & Norwegian Breast Cancer Society (#207819) and Wellcome Leap’s Dynamic Resilience Program (jointly funded by Temasek Trust) (#104617). The Fang Laboratory is also supported by Cure Alzheimer’s Fund (#282952), HELSE SØR-ØST (#2020001, #2021021, and #2023093), the Research Council of Norway (#262175 and #334361), Molecule AG/VITADAO (#282942), the NordForsk Foundation (#119986), the National Natural Science Foundation of China (#81971327), Akershus University Hospital (#269901, #261973, and #262960), the Civitan Norges Forskningsfond for Alzheimers sykdom (#281931), and the Czech Republic-Norway KAPPA programme (#TO01000215). C.A.L. was supported by the NCI (R37CA237421, R01CA248160, and R01CA244931). Work in the E.F.F. and R.H.H. labs is supported by funding from the European Union’s Horizon Europe research and innovation program through the MSCA-Doctoral Network NADIS (no. 101073251). The graphical abstract was prepared using BioRender software (https://biorender.com/).

## Author contributions

Conceptualization and experimental design, E.F.F.; experimental investigation, H.-L.W., J.Z., M.J.L.-D., S.L., and S.-q.Z.; writing – original draft, H.-L.W. and J.Z.; writing – review and editing, E.F.F., J.Z., S.L., Z.H., C.A.L., and R.H.H.; resources, E.F.F.; funding acquisition, E.F.F.; supervision, E.F.F. All authors read the manuscript and provided their final approval of the content.

## Declaration of interests

E.F.F. is a co-owner of Fang-S Consultation AS (organization number 931 410 717) and NO-Age AS (organization number 933 219 127); he has an MTA with Lmito Therapeutics Inc. (South Korea), a CRADA arrangement with ChromaDex (USA), and a commercialization agreement with Molecule AG/VitaDAO; and he is a consultant to MindRank AI (China), NYO3 (Norway), and AgeLab (Vitality Nordic AS, Norway). In the past three years, C.A.L. has consulted for Astellas Pharmaceuticals, Odyssey Therapeutics, Third Rock Ventures, and T-knife Therapeutics and is an inventor on patents pertaining to Kras-regulated metabolic pathways, redox control pathways in pancreatic cancer, and targeting of the GOT1-ME1 pathway as a therapeutic approach (US Patent No: 2015126580-A1, 05/07/2015; US Patent No: 20190136238, 05/09/2019; International Patent No: WO2013177426-A2, 04/23/2015).
